# Lifestyle modifications: coordinating the tRNA epitranscriptome with codon bias to adapt translation during stress responses

**DOI:** 10.1186/s13059-018-1611-1

**Published:** 2018-12-27

**Authors:** Cheryl Chan, Phuong Pham, Peter C. Dedon, Thomas J. Begley

**Affiliations:** 1 Singapore-MIT Alliance for Research and Technology, 1 CREATE Way, 138602 Singapore; 2 Department of Biological Engineering, Massachusetts Institute of Technology, Cambridge, MA 02139 USA; 3 The RNA Institute, College of Arts and Science, University at Albany, SUNY, Albany, NY 12222 USA

## Abstract

Cells adapt to stress by altering gene expression at multiple levels. Here, we propose a new mechanism regulating stress-dependent gene expression at the level of translation, with coordinated interplay between the tRNA epitranscriptome and biased codon usage in families of stress-response genes. In this model, auxiliary genetic information contained in synonymous codon usage enables regulation of codon-biased and functionally related transcripts by dynamic changes in the tRNA epitranscriptome. This model partly explains the association between synchronous stress-dependent epitranscriptomic marks and significant multi-codon usage skewing in families of translationally regulated transcripts. The model also predicts translational adaptation during viral infections.

**Electronic supplementary material:**

The online version of this article (10.1186/s13059-018-1611-1) contains supplementary material, which is available to authorized users.

## Introduction

The ‘central dogma’ defines the ‘what’ of biology—genes are transcribed into messenger RNAs that are translated into proteins. But it says nothing about the ‘when’ or ‘how much’ of gene expression. The application of systems-level ‘-omic’ technologies has led to the discovery of information-rich and combinatorial scheduling systems for gene expression involving dozens of enzyme-catalyzed chemical modifications of DNA, RNA and proteins—the epigenome and epitranscriptome. Here, we explore the evidence for a mechanism of translational control of gene expression in which the earliest and best-known RNA ‘marks’—the dozens of modified nucleosides in the transfer RNA (tRNA) epitranscriptome—interact with what amounts to families of transcripts that possess skewed usage patterns for many codons to fast-track production of survival proteins during stress.

The scheduling of gene expression at the level of transcription is well established in the literature of transcription factors, splicing, and messenger RNA (mRNA) stability, among many other mechanisms, including the complicated function of micro-RNAs (miRNAs), Piwi-interacting RNA (piRNA), small nuclear RNA (snRNA), long non-coding RNA (lncRNA), tRNA-derived stress-induced RNA (tiRNA), and tRNA-derived RNA fragment (tRF) RNA [1]. The idea of heritable mechanisms for scheduling transcription in eukaryotic cells has more recently emerged in the form of the epigenome, with unique DNA and histone protein modification patterns in each cell type determining which genes are transcribed [2, 3]. However, there has been a long-standing dilemma posed by the observation that the correlation between changes in levels of mRNA and protein can be relatively poor, with correlation coefficients on the order of 0.4 [4, 5]. The correlation in changes in mRNA and protein levels improves somewhat when systematic delays between transcription and translation are considered [6], but other mechanisms must exist to account for changes in protein levels that do not reflect the abundance of their mRNA. Among the post-transcriptional mechanisms for scheduling gene expression, protein degradation mechanisms [7] have been more clearly delineated than translational mechanisms, with the latter largely focused on translation initiation, efficiency, and fidelity rather than scheduling [8].

**Fig. 1:**
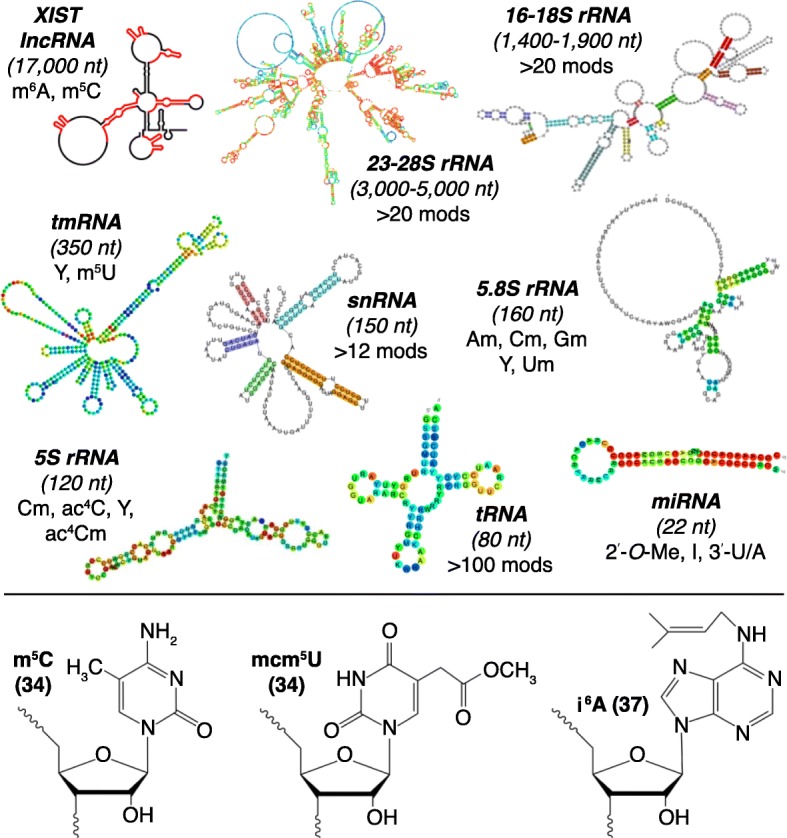
Prototypical RNA species and their modifications. All types of RNA are modified; structures for three of more than 120 modifications are shown in the *lower panel*. RNA secondary structures were adapted from the following sources: XIST long non-coding RNA (lncRNA) [82]; 5S and 5.8S rRNA, tRNA, microRNA (miRNA), small nuclear RNA (snRNA), transfer-messenger RNA (tmRNA), 16-18S, 23-28S rRNA [83]. *Abbreviations*: *ac*^*4*^*C* N4-acetylcytidine, *ac*^*4*^*Cm* N4-acetyl-2’-O-methylcytidine, *Am* 2’-O-methyladenosine, *Cm* 2’-O-methylcytidine, *Gm* 2’-O-methylguanosine, *I* inosine, *i*^*6*^*A* N6-isopentenyladenosine, *mcm*^*5*^*U* 5-methoxycarbonylmethyluridine, *m*^*5*^*C* 5-methylcytidine, *m*^*5*^*U* 5-methyluridine, *m*^*6*^*A* N^6^-methyladenosine, *nt* nucleotide, *3′-U/A* 3′-uridylation/adenylation, *2’-O-Me* 2’-O-methylation, *Um* 2’-O-methyluridine, *Y* pseudouridine

**Fig. 2:**
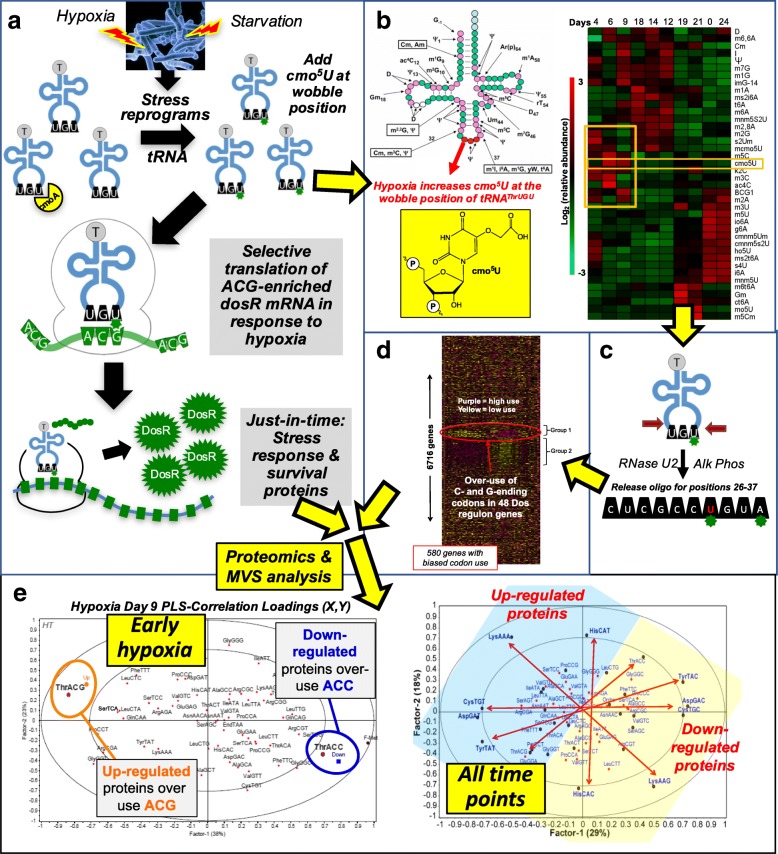
tRNA reprogramming and codon-biased translation of stress-response proteins. **a** Model illustrating the effects on *Mycobacterium bovis* BCG of hypoxia encountered during infection. Hypoxia induces expression of 48 proteins in the Dos regulon that causes the cell to become dormant. **b** The tRNA epitranscriptome consists of over 120 post-transcriptionally inserted modified ribonucleosides. During hypoxia, the relative quantities of 40 tRNA modifications (rows in heat map) change as a function of time during the response to the stress (days 0–21; columns in heat map) and again during O_2_ resuscitation (days 22–24). In early hypoxia (day 9), the wobble position of tRNAThr^UGU^, which reads the codon ACG, switches from 5-methoxyuridine (mo^5^U) to 5-oxyacetic acid uridine (cmo^5^U; structure shown). **c** RNase/LC-MS maps cmo^5^U to the wobble of tRNAThr^UGU^. **d** Families of response genes are organized by biased use of synonymous codons. The heat map shows over-use (*purple*) and under-use (*yellow*) of 62 codons (columns) across all genes (rows) in BCG. The gene for DosR, the master regulator of the 48-gene Dos regulon, over-uses the ACG codon and under-uses ACC, the most common Thr codon. **e** Codon analysis of proteomics data shows that > 80% of proteins upregulated in early hypoxia use ACG to code for Thr, whereas downregulated proteins are enriched in the so-called ‘optimal’ codon for Thr, ACC. Evidence that the auxiliary information in the genetic code is utilized for regulatory purposes is supported by examining codons associated with highly upregulated and downregulated proteins across all time-points of hypoxia in BCG. Pairs of synonymous codons are differentially enriched in upregulated and downregulated proteins, with the codon enrichments defining functional gene families. *Alk phos* alkaline phosphatase, *PLS* partial least squares, *Thr* threonine

Enter the epitranscriptome—now defined as the set of modified ribonucleotides in all forms of RNA, coding and non-coding (Fig. 1). While more than 120 different modifications have been cataloged in transfer RNA (tRNA) and ribosomal RNA (rRNA) over the past half-century, the term ‘epitranscriptome’ was first applied in 2012 to the subset of modifications in mRNA, with the observation of dynamic changes in the levels of N^6^-methyladenosine (m^6^A) in different transcripts [9]. It is now recognized that virtually every form of RNA contains modified ribonucleotides (Fig. 1), and these have been extensively reviewed elsewhere [10–16]. Significant advances have been made in defining the role of individual modifications in regulating RNA stability and translation rate, efficiency, and fidelity [10, 17–37]. However, the most mechanistically detailed models for systems-level functions of RNA modifications have arisen from studies of stress reprogramming of the tRNA epitranscriptome, and were further supported by computational studies of codon usage and the analysis of codon pausing using ribosome profiling of cells deficient in or ‘addicted’ to tRNA modification enzymes [21–23, 25, 27–29, 34, 38–40]. Based on a growing literature, we propose a model for scheduling protein synthesis of many crucial stress-response proteins involving coordinated interactions between the tRNA epitranscriptome and a select group of transcripts possessing skewed usage patterns for many codons (Fig. 2a). Supporting this ‘translational control model’, stress-induced changes in tRNA modifications that regulate the translation of codon-biased transcripts have been observed in bacteria, yeast, and mouse cells [21–23, 27–29, 34, 38, 39]. Defects in tRNA modifications and specific tRNA-modifying enzymes have also been shown to coordinately regulate the synthesis of groups of proteins from codon-biased genes in human cancer models and many other cell types [21–23, 25, 27–29, 34, 38–41]. Here, we consider the general principles of how dynamic changes in the tRNA epitranscriptome can coordinately regulate the translation of codon-biased transcripts. We note that this model is not applicable for all the expressed transcripts in a cell, but the ~ 5 to 10% that have statistically significant deviations in codon-usage patterns for multiple codons, relative to genome averages. We primarily illustrate detailed features of our model with our work on the mycobacterial response to hypoxic stress. We conclude by describing the potential implications of the tRNA epitranscriptome and codon-usage patterns for viral infections.

## Different stresses uniquely reprogram dozens of tRNA modifications

All cells possess ~ 30–50 post-transcriptional modifications of ribonucleosides in RNA—known as the epitranscriptome (see Fig. 1 for sample structures and Fig. 2b for names). With 10% of the ~ 80 nucleotides in tRNA modified from A, C, G, and U, tRNA is the most heavily decorated form of RNA, greatly exceeding the diversity and frequency of modification levels in mRNA, rRNA, and other forms of RNA. Many of the > 120 modified ribonucleosides identified to date [42, 43] influence tRNA stability [17, 18, 20] and translational speed [30] and fidelity [19, 26, 31–33, 36]. For example, 1-methyladenosine (m^1^A) at position 58 of both yeast and human tRNAiMet has been reported to be crucial for its stability [17, 18], whereas ‘restrictive’ modifications at the tRNA anticodon wobble uridine position 34 in prokaryotes and eukaryotes, such as 5-methylaminomethyl-2-thio (mnm^5^s^2^) and 5-methoxycarbonylmethyl-2-thio (mcm^5^s^2^), respectively, enhance the translation of the cognate codons by enhancing ribosome A-site occupancy, stacking interactions for codon–anticodon interactions, and prevent frame shifting [19, 26, 36]. Similarly, modifications at tRNA position 37 adjacent to the anticodon loop, such as N6-threonylcarbamoyladenosine (t^6^A) and N6-isopentenyladenosine (i^6^A), are thought to prevent frame shifting, thereby enhancing the fidelity of translation [19, 33, 36].

An important enabler in the study of epitranscriptomics was the development of sensitive mass-spectrometry-based ‘-omic’ approaches to simultaneously identify and quantify the full set of modified ribonucleosides [44–47], which complements published studies quantifying individual modification systems and their link to codon-biased translation [22, 23, 38, 40, 48–51]. Using a sensitive method based on liquid chromatography–tandem mass spectrometry (LC-MS/MS) [44, 52], it was found that cells respond to different stresses by uniquely changing the relative quantities of ~ 30–50 tRNA modifications. It was discovered that tRNA modifications behave in a coordinated manner to control gene expression at the level of translation during cell stress responses—the first facet of the translational control model (Fig. 2a). Subsequent studies profiling the tRNA modification landscape from bacteria [34] to yeast [21, 27, 39, 53] to mammalian cells [29] subjected to a variety of stressors revealed the generality of tRNA epitranscriptome reprogramming as part of the cell-stress response. For example, mycobacteria respond to hypoxic stress by gradually shifting from logarithmic growth to a non-replicative, drug-resistant dormant state over several weeks. The heat map in Fig. 2b illustrates the tRNA modification reprogramming dynamics in response to hypoxia—unique patterns of increased (red) and decreased (green) levels of ~ 40 tRNA modifications (rows) at each time-point (columns) during hypoxia (days 0–21) and subsequent rescue by re-aeration (days 22–24). A striking ‘oxic’ pattern of tRNA modification changes can be observed common to both pre-hypoxia (day 0) and during re-aeration (day 24) conditions, but not during hypoxia (days 4–21) [34]. These tRNA modification signatures are > 80% predictive of specific chemical stresses in yeast and hence contain significant information relevant to the underlying stress-response mechanisms [27, 39]. How this population-level information, which has been observed by others [54, 55], can be linked to mechanisms governing translation became apparent when stress-regulated modifications were mapped to specific tRNAs.

## Stress reprograms specific wobble modifications in specific tRNA isoacceptors

The link between stress-specific modification ‘reprogramming’ and the regulation of protein translation became clearer through mapping the stress-altered ribonucleosides to specific tRNA isoacceptors (Fig. 2c) [44]. By leveraging another mass-spectrometry (MS)-based approach similar to that applied to proteomics—MS-sequencing of specific tRNAs [44, 45]—the exact location of stress-altered ribonucleosides could be mapped in individual tRNA molecules. For example, the 5-oxyacetic acid uridine (cmo^5^U) modification observed to increase as part of the early response to hypoxia (day 6–9) in mycobacteria (Fig. 2b, yellow box) was mapped to the wobble position of tRNA^Thr^ that reads the ACG codon (Fig. 2c). Using a systems-level codon analytics tool [56], this observation led to a key discovery—that cognate codons of tRNAs possessing stress-altered modifications, such as cmo^5^U-dependent ACG, are enriched in some families of stress-response genes. Furthermore, these ACG-enriched transcripts are also over-utilizing other codons. Our findings support the idea that the auxiliary genetic information found in the form of synonymous codons is utilized during stress responses. In addition, we and others have predicted that there exists a system of differential use of subsets of the genetic code, which can also be defined as using codon bias as auxiliary genetic information [28, 34, 38, 57]. In many cases, one of the synonymous codons specific to each amino acid is differentially enriched in genes coding for proteins that are translationally regulated by epitranscriptomic marks during a specific stress (Fig. 2d, e). This same phenomenon linking stress-dependent changes in the wobble modifications of specific isoacceptors or modification enzymes to biased use of the cognate codon in stress-response genes has been observed in diverse cell types by various groups [21–23, 25, 27–29, 34, 38–40].

## Building the case for codon-biased translation—Families of stress-response genes are distinguished by biased use of synonymous codons

**Fig. 3:**
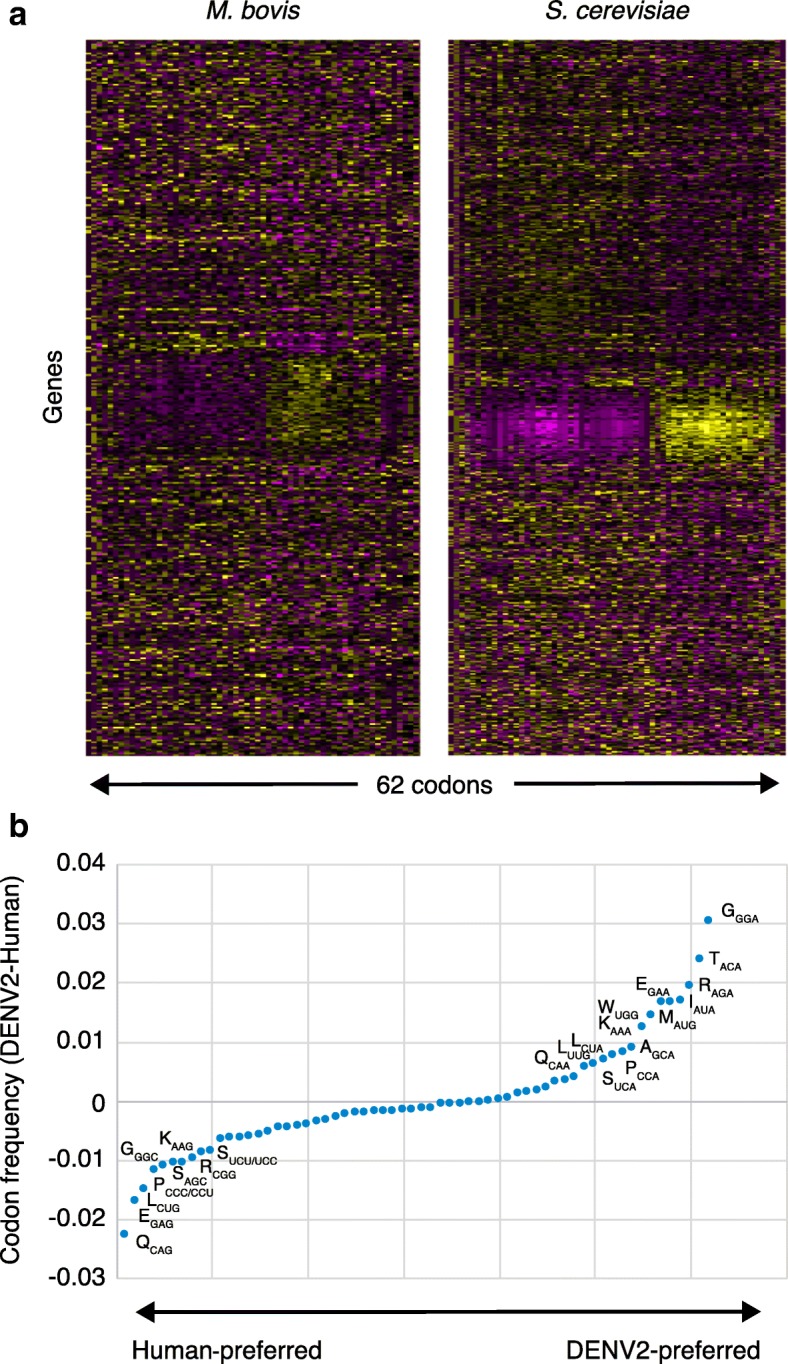
Patterns of synonymous codon usage define families of stress-response genes and might predict epitranscriptomic responses to viral infections. **a** The idea that translation regulation uses auxiliary genetic information in the form of codon bias arose by linking systems-level analyses of stress-induced proteomic upregulation and downregulation [21, 28, 34] with codon analytics [28, 38]. The heat maps shown here are examples of genome-level application of a codon-counting algorithm and visualization approaches [28, 34] to the genomes of *Mycobacterium bovis* BCG and *Saccharomyces cerevisiae*. The maps show over-use (*yellow*) and under-use (*purple*) of 62 codons (columns) across all genes (rows). For each organism, clusters represent groups of genes that have distinctly different codon-usage patterns compared with genome averages, with two opposing groups of genes identified in humans. As shown in Fig. 2d, the smaller of two clusters of codon-biased genes in *M. bovis* BCG consists of the DosR regulon of 48 genes that control the response to hypoxic stress [34]. **b** Widely differing codon-usage patterns in the human genome and a representative dengue serotype 2 genome (DENV2) could predict reprogramming of the host cell tRNA epitranscriptome both to accommodate the codon mismatch in the viral RNA genome and to respond to the stress of viral infection. Codon frequency data were generated using the web-analysis interface on the published Codon Utilization Tool (CUT) [56] and the publicly available human and dengue sequence information (Human refseq_hg38 and dengue virus 2, complete genome NCBI Reference Sequence: KM204118.1), together with human and dengue frequency data found in Additional file 1. The analysis shows that the DENV2 genome is biased toward A-ending codons, whereas the human genome is biased toward G- and C-ending codons. This leads to the testable hypothesis that DENV2 infections will cause changes in the host cell tRNA pool, both modifications and copy numbers, to simultaneously accommodate translation of the viral mRNA and facilitate translation of codon-biased host stress-response genes

The idea that there is a system of biased codon usage in groups of transcripts represents the second facet of the translational control model (Fig. 2a). While obviously affected by the GC-content of the genome of an organism, the biological functions of biased codon usage have been the subject of numerous studies over the past several decades and include proposals for a relationship between codon usage in 5′-mRNA secondary structures and translation initiation, modest correlations between enrichment with ‘optimal’ codons and the abundance of cognate tRNA isoacceptors in the pool, and differential effects of codon usage on translation elongation rate, translation fidelity and, likely, protein folding [22, 30, 58–68]. Building on the observation that genes encoding amino acid biosynthesis pathways are enriched with rare codons that are read by tRNAs that remain highly charged during amino acid starvation [64, 69], several groups have proposed the general idea of a role for biased codon usage in families of stress-response genes [21–23, 25, 27–29, 34, 38–40]. This idea was supported by gene-specific analysis of codon-usage patterns across eukaryotic and prokaryotic genomes using an algorithm to quantify usage frequencies of each of the 62 codons in each gene in an organism relative to genome-average values. The genomes of *Mycobacterium bovis* BCG and the budding yeast *Saccharomyces cerevisiae* (Fig. 3; Additional file 1) [34, 38, 56] illustrate the point that there are hundreds of genes with biased codon-usage patterns (Fig. 3, ‘over-used’ codons in yellow, ‘under-used’ codons in purple) relative to genome averages. It is important here to clarify the difference between ‘over-used’ codons and the so-called ‘optimal’ codons—codons that are the most frequently used based on a genome average, with a high abundance of the decoding tRNAs in the pool. There is a widely held view that the most abundantly expressed proteins are the result of efficient translation of genes enriched in ‘optimal’ codons that match the most abundant tRNAs in the pool [65, 66]. This has led to the current practice of codon optimization for foreign genes expressed in a different host cell, in which the codons in the foreign gene are replaced with the ‘optimal’ codons of the host cell (e.g., see [70]). However, ‘optimal’ codons only reflect very specific high-abundance proteins expressed in unstressed cells grown under optimal conditions, with the many codon-biased genes not falling into the category of being highly expressed [56, 71].

In contrast to classical codon optimality and many of the proposed functional models for genomic codon bias, a number of studies in eukaryotes and prokaryotes have now shown that groups or families of genes involved in stress responses systematically over-use and under-use specific ‘non-optimal’ synonymous codons. This is illustrated by the observation that the 48 genes in the DosR regulon in *M. bovis* BCG, which are essential for survival under hypoxic stress [34, 72], are enriched in G- and C-ending codons [34] (Figs. 2d and 3). More examples of this idea of codon-biased stress-response genes are provided by amino acid biosynthesis genes that are crucial during amino acid starvation in *Escherichia coli* [64, 69], Elongator-dependent translation of codon-biased families of cell-division genes [22, 23], and wobble uridine U34 modification-dependent regulation of glycolytic genes by codon-biased expression of the *HIF1A* gene in melanoma cells [40], as well as tRNA modification-dependent and codon-dependent regulation of the oncoprotein DEK, which regulates the IRES-dependent translation of the transcription factor LEF1 in breast cancer metastasis models [41]. These observations of codon bias in gene families and global regulators further support the idea that some transcripts will be more efficiently translated under specific stress conditions through stress-induced changes in the tRNA epitranscriptome in order to optimize translation and levels of the appropriate protein response systems and networks (Fig. 2). The evidence for this model is discussed below.

## Stress-reprogrammed tRNAs are required for translation of codon-biased mRNAs encoding stress-response proteins in bacteria, yeast, and mammals

That there is a mechanistic link between the stress-reprogrammed tRNA epitranscriptome, the existence of gene-specific codon-usage patterns, and selective translation of codon-biased mRNAs for stress-response genes (Fig. 2a) is borne out in a variety of studies in bacteria, yeast, and human cells [21–23, 25, 27–29, 34, 38–40]. The most striking illustrations of this mechanism arise from linked analysis of stress-induced changes in the transcriptomes, epitranscriptomes, and proteomes in yeast and bacteria [21, 28, 34]. In the case of *M. bovis* BCG subjected to hypoxic stress, multivariate statistical analysis of proteomic data revealed that pairs of synonymous codons were differentially enriched in genes for proteins that were upregulated or downregulated across the hypoxia time-course (Fig. 2e). For example, the early-response (day 9) of mycobacteria to hypoxic stress involves upregulating proteins from genes enriched with the Thr codon ACG and downregulating proteins from genes enriched with the synonymous Thr codon ACC (Fig. 2e, left panel). That the expression of codon-biased gene families is controlled mainly at the level of translation and not transcription was established by correcting the proteomic analyses for mRNA expression and protein abundance differences [21, 28, 34, 38]. We have further observed that this dichotomous pattern of differentially used codons generalizes in all sampled time-points of the mycobacteria hypoxic stress response (Fig. 2e)—under a specific stress condition, most upregulated proteins are enriched with one synonymous codon, whereas downregulated proteins are enriched with the partner synonymous codon. It is important to point out that hypoxic stress increased the translation of proteins from genes enriched with non-‘optimal’ codons ACG (Thr), CTA (Leu), GCG (Ala), and GGA (Gly), whereas their synonymous partners ACC (Thr), CTT (Leu), GCT (Ala), and GGT (Gly) were all overrepresented in downregulated proteins in hypoxia [34].

In some cases, an organism will use an optimal codon more than expected and pair it with low-usage codons in a transcript [21, 28, 38]. Alkylation stress by exposure to methyl methanesulfonate (MMS) increased Trm9-dependent wobble uridine 5-methoxycarbonylmethyl (mcm^5^) and mcm^5^s^2^ modifications linked to tRNA^Arg^(UCU) and tRNA^Glu^(UUC), with increased translation of mRNAs enriched in the cognate AGA and GAA codons, respectively [27, 38, 53]. Interestingly, however, the Trm9-dependent codons AGA (Arg) and GAA (Glu) are enriched in DNA damage and cell-cycle control genes crucial to surviving alkylation stresses [28, 38]. In addition, the non-optimal codons GAC (Asp), ATC (Iso), TAC (Tyr), AAG (Lys), and TTC (Phe) are found paired with AGA and GAA on the transcripts whose translation is linked to 5-methoxycarbonylmethyluridine (mcm^5^U) and 5-methoxycarbonylmethyl-2-thiouridine (mcm^5^s^2^U) [38]. The identified codon-biased genes are among those clustering in the heat map of codon usage in the *S. cerevisiae* genome shown in Fig. 3. Coupled with the biased use of synonymous codons in families of stress-response genes, this more than pair-wise use of one synonymous codon from a set to control protein upregulation and downregulation represents a systematic repurposing of the auxiliary information tied to the genetic code for adaptation and survival in a changing environment.

This association between the stress-altered epitranscriptome and translation of codon-biased stress-response genes has also been observed in two forms of yeast, the nematode *Caenorhabditis elegans*, and human [21–23, 25, 27–29, 34, 38–40]. For example, *S. cerevisiae* responded to H_2_O_2_-induced oxidative stress by increasing Trm4 methyltransferase-dependent wobble 5-methylcytidine (m^5^C)-modification in tRNA^Leu^(CAA), which resulted in the selective translation of mRNA from genes enriched in the cognate TTG codon [21]. Similarly, mitosis and cytokinesis in the fission yeast *Schizosaccharomyces pombe* is controlled by Elongator-mediated synthesis of mcm^5^s^2^ in tRNAs recognizing AA-ending codons, with these codons enriched in three different groups of proteins, including proteins involved in cell division [22, 23]. More recently, Close and colleagues demonstrated that the carcinogenic and drug-resistant phenotype of human melanoma cells required Elongator-dependent translation of codon-biased mRNAs, including the stress-response mediator *HIF1A* [40]. These results are consistent with a general mechanism for enhancing or scheduling translation of proteins needed by the cell to mount an appropriate response to specific stress conditions.

Further support that stress-regulated tRNA modifications are directly linked to expression of codon-biased survival proteins has emerged in the previously described studies in yeast, *C. elegans*, and human [21–23, 25, 27–29, 38, 40]. In all cases, cells lacking the tRNA-modifying enzymes did not show the specific, modification-dependent codon-biased translation and had distinct proliferative defects [21–23, 25, 27–29, 38, 40]. In yeast, this rendered the cells hypersensitive to killing by the stresses [21, 28, 39], whereas in human melanoma cells, this reduced the tumorigenicity and drug resistance of the cancer cells [40]. For example, loss of Trm4 in *S. cerevisiae* prevented m^5^C formation at the wobble position of tRNA^Leu^(CAA), abolished selective translation of mRNA from genes enriched in the TTG codon, and resulted in hypersensitivity of cells to H_2_O_2_ [21]. Similarly, deletion of Trm9 prevented formation of its product mcm^5^U in tRNA^Arg^(UCU), with a concomitant enhanced sensitivity to MMS [28, 38, 67].

In mammals, a good example of a specific tRNA epitranscriptomic mark regulating the translation of codon-biased mRNAs has been identified in the synthesis of the stress-important selenoproteins [29], which include the H_2_O_2_-detoxifying glutathione peroxidase (Gpx) and thioredoxin reductase (TrxR) enzymes. The approximately 25 known selenoproteins contain the 21st amino acid selenocysteine (Sec). Notably, Sec does not contain a dedicated codon, but instead uses a recoded stop-codon (UGA) along with specific wobble U modifications in tRNA^SER(SEC)^ and many other factors to promote the incorporation of Sec into the growing peptide chain [73–75]. Stress-responsive selenoproteins are an excellent example of our translational control model, albeit very specific, as corresponding transcripts have at least twice as many stop codons as expected (i.e., biased codon usage) and require increased 5-methoxycarbonylmethyl-2’-O-methyluridine (mcm^5^Um) modifications in tRNA to increase the levels of some Gpx and TrxR enzymes in response to H_2_O_2_ [29, 56]. The observations of Close and coworkers in human melanoma cells and breast cancer cells provide two examples of mechanisms where tRNA modification-enzyme-dependent codon-biased translation of a master regulator controls network-based responses [40, 41]. Although the observations of codon-dependent translational control of HIF1α and DEK are not examples of broad translational control itself, they do nevertheless control broad transcriptional networks. Combined, these findings further support the notion of coordinated interactions between the tRNA epitranscriptome and biased codon usage to enhance translation of survival proteins.

## Implications of a system linking the epitranscriptome to a code of codons—Viral infections

One example of how the proposed epitranscriptome-based gene regulation model can be ‘translated’ to other arenas involves viral infections. Viruses depend on the translational machinery of the host cell for survival and replication, which poses a significant problem in light of the highly different codon-usage patterns in the viral and host-cell genomes. Consider, for example, RNA-based Flaviviruses that rely on the translational machinery of the host for immediate synthesis of viral proteins essential for replication and survival after infection. A comparison of the codon usage frequencies in the human genome and the ~ 11 kb genome of a representative Flavivirus—dengue virus serotype 2 (DENV2)—showed that the viral RNA genome is heavily biased toward A-ending codons, whereas the human genome is biased toward C- and G-ending codons (Fig. 3b), in spite of the similar GC content of the two genomes (human 42% GC versus DENV2 46% GC). This raises a potential problem for the virus, in part when it needs to translate what are rare codons for the host and for which the cognate tRNAs are present in the pool at low levels or with inappropriate modifications. This mismatch between codon usage and the tRNA pool could lead to stalled translation, mistranslation, and proteotoxic stress, which would be detrimental to the virus. In addition to potentially optimizing the levels of specific host tRNA isoacceptors as a strategy by the virus to ensure efficient translation of its unique genome, our translational control model predicts the need to reprogram the host tRNA epitranscriptome, particularly at the wobble uridine bases to enhance decoding of what would normally be considered rare A-ending codons for the human host.

An intriguing feature of the dengue genome raises questions about the ability of the virus to co-opt the host translational machinery to facilitate translation of viral proteins in the face of codon mismatch. Among the ten proteins coded by the dengue genome, the NS5 protein is an RNA methyltransferase that catalyzes formation of 7-methylguanosine (m^7^G) and 2′-O-methyladenosine (Am) in the GpppA cap added to the viral RNA, and additionally forms Am throughout the viral genome [76]. In vitro reactions revealed that NS5 can methylate human rRNA [76], raising the possibility of a viral methyltransferase that could directly coordinate reprogramming of the host translational epitranscriptome to enhance translation of the viral genome. Recent studies using a similar LC-MS/MS approach to ours but applied instead to the analysis of non-size-selected, hydrolyzed total cellular RNA and viral RNA isolated from RNA-virus-infected cell cultures showed changes in RNA post-transcriptional modifications (PTMs) occurring following viral stress on host cells [54, 77]. Future studies are needed to carefully deconvolute this initial landscape of PTMs in terms of their site, function, and interplay in the cell-stress response. Additionally, the promiscuity of the dengue NS5 methyltransferase for both rRNA and tRNA substrates is reminiscent of known dual-specificity RNA-modifying enzymes such as pseudouridine synthase RluA and RNA methyltransferase RlmN in *E. coli* that modify both tRNA and rRNA bases [78, 79]. Could this be a common mechanism to synchronize changes across epitranscriptomes? Recent work demonstrating the synchrony between cytosolic and mitochondrial translation [80] raises yet another interesting possibility of crosstalk between both epitranscriptomes that can build on the translation control model we present here. With a direct-acting methyltransferase and known modulatory effects on the cellular metabolism of the host [81], the Flavivirus–human host-cell infection model presents an attractive system to probe the ribonucleome and epitranscriptome reprogramming of the host cell in response to viral stress and to gain insights into potentially important mechanisms.

## Concluding remarks

Our proposed mechanism for translational adaptation involving coordinated interplay between the tRNA epitranscriptome and biased codon usage represents a complicated interaction among diverse systems and is well supported by observations in prokaryotes and eukaryotes. In addition to generating numerous testable hypotheses concerning controlling gene expression at the level of translation—such as using codon bias as a predictor of epitranscriptome dynamics—this system has important implications for synthetic biology in the form of genetic tools to tune the pool of tRNA molecules and the dozens of programmable tRNA modifications, for predicting translational adaptation during viral infections, and for expression of foreign genes in cells.

## Additional file

Additional file 1: **Table S1.** Tabulated codon usage frequencies for dengue virus and human genomes. (XLSX 15 kb)
